# Biosensing Systems Based on Graphene Oxide Fluorescence Quenching Effect

**DOI:** 10.3390/mi14081522

**Published:** 2023-07-28

**Authors:** Antonella Battisti, Sangram Keshari Samal, Dario Puppi

**Affiliations:** 1 NEST, Istituto Nanoscienze-CNR and Scuola Normale Superiore, p.zza San Silvestro 12, I-56127 Pisa, Italy; 2 Laboratory of Biomaterials and Regenerative Medicine for Advanced Therapies, ICMR-RMRC, Bhubaneswar 751023, Odisha, India; sksamalrec@gmail.com; 3 BIOLab Research Group, Department of Chemistry and Industrial Chemistry, University of Pisa, UdR INSTM Pisa, Via Moruzzi 13, I-56124 Pisa, Italy; dario.puppi@unipi.it

**Keywords:** graphene oxide, fluorescence biosensors, fluorescence quenching, fluorescence graphene oxide sensors

## Abstract

Graphene oxide (GO) is a versatile material obtained by the strong oxidation of graphite. Among its peculiar properties, there is the outstanding ability to significantly alter the fluorescence of many common fluorophores and dyes. This property has been exploited in the design of novel switch-ON and switch-OFF fluorescence biosensing platforms for the detection of a plethora of biomolecules, especially pathological biomarkers and environmental contaminants. Currently, novel advanced strategies are being developed for therapeutic, diagnostic and theranostic approaches to widespread pathologies caused by viral or bacterial agents, as well as to cancer. This work illustrates an overview of the most recent applications of GO-based sensing systems relying on its fluorescence quenching effect.

## 1. Introduction

**Figure 1:**
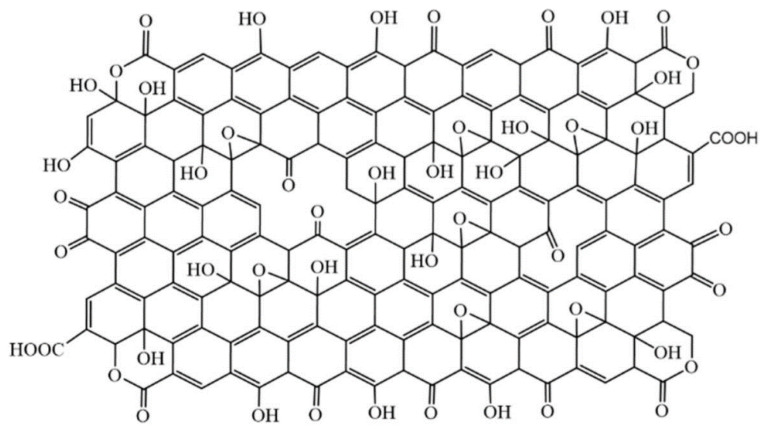
Proposed structure for graphene oxide (GO). Reproduced from ref. [8]. © 2023 by the authors. Licensee MDPI, Basel, Switzerland (CC BY 4.0).

Carbon nanomaterials are versatile entities that have been proven to be valid in many different fields, thanks to their peculiar physical, chemical, optical, thermal and electrical behavior [1]. In recent years, potential applications of carbon nanomaterials have been explored in many contexts, such as smart material development, energy storage, sensing devices, and so on. Even if graphene recently became the undiscussed leader of this class of materials, its oxidized version, graphene oxide (GO), firstly obtained by the harsh oxidation of graphite in a strongly acidic environment [2], is playing a major role in theranostics, drug delivery, biosensing, anticancer therapy and other biomedical related applications [3,4,5]. This is because GO preserves many of the peculiar features of graphene, such as optical transparency, flexibility, and cell compatibility, but it also has distinctive and tunable physicochemical properties that give this material a central role in the production of miniaturized biosensors. Indeed, GO sheets expose many oxygen-containing functional groups (mainly epoxides and hydroxyls on the surfaces [6], but also carboxyls and lactones on the edges and defects [7,8]) that induce good water solubility, chemical reactivity, and the ability to establish covalent interactions with several molecules and biomolecules (Figure 1) [9].

**Figure 2:**
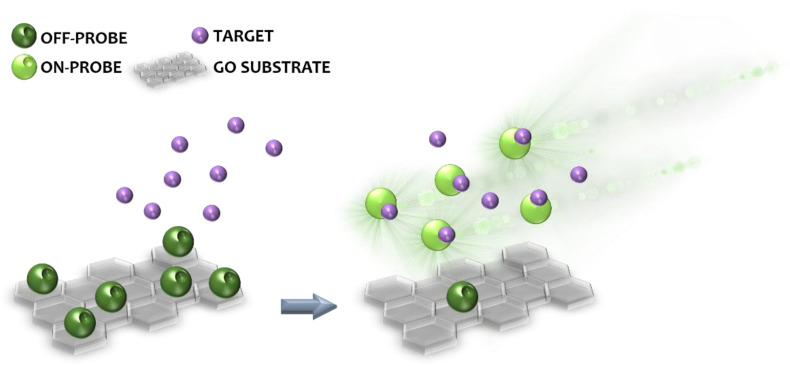
The quenched fluorescent probe (OFF-PROBE) adsorbed on GO surface (**left**) undergoes switch-ON effect upon target binding (**right**) and emits fluorescence (ON-PROBE).

These features have been extensively explored to produce novel sensors and biosensors for applications in many areas, including the environmental [10] and biomedical [11] fields. Biosensors are made of a receptor and a transducer, where the receptor can be a biomolecule that interacts with the targeted analyte and the transducer offers sensing information that transfers the electrical, chemical or optical information into a detectable signal [12,13]. Among other sensing mechanisms, based e.g., on laser desorption/ionization mass spectrometry (LDI-MS) [14], surface-enhanced Raman spectroscopy (SERS) [15], and electrochemistry [16], fluorescence-based GO biosensors gained increasing attention thanks to the ability of GO to quench the fluorescence of labeling dyes and fluorophores of biosensing probes [17]. Basically, fluorescent probes can be adsorbed on the surface of GO, which significantly quenches their fluorescence; in the presence of the target molecule, the probe leaves the GO surface due to the establishment of strong probe–target interactions and the fluorescence signal is recovered (switch-ON effect), revealing the presence of the target molecule (Figure 2). This work intends to review the latest advances in the application of fluorescence quenching GO-based sensing systems for the detection of viruses, bacteria, and cancer cells, for biomedical purposes.

## 2. The Role of GO in Fluorescence Detection

Driven by their potential impact on energy technology, graphene and GO have been widely characterized for the energy/charge transfer between them and other donors or acceptors. GO is commonly considered a fluorescence quencher for fluorophores [18,19,20] and fluorescent biomolecules [21,22,23], mainly due to energy transfer, electron transfer and non-radiative dipole–dipole coupling channels [24]. Albeit fluorescence enhancement effects may occur under certain conditions (as described at the end of this section), GO is typically considered a fluorescence quencher and is used as such for sensing applications. 

**Figure 3:**
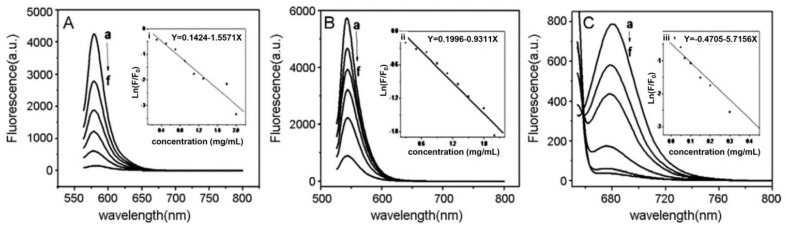
Steady-state fluorescence spectra of dyes (10^−5^ mg/mL in water) in presence of different concentrations of GO as a quencher (curves a–f: a: lowest GO concentration, f: highest GO concentration). (**A**) rhodamine B, (**B**) eosin, (**C**) methylene blue. (i–iii) linear relationship between the corresponding dye fluorescence (a.u.) and GO concentration (mg/mL). Reproduced with permission from ref. [17]. © 2023 Elsevier B.V.

Fluorescence quenching is basically a collective name referring to any process that decreases the fluorescence intensity of a fluorophore. After the absorption of a photon, quenching phenomena can promote non-radiative paths for the fluorophore relaxation from the excited state back to the ground state. This can occur by different mechanisms such as energy transfer, electron transfer, excited-state reactions, molecular conformational changes, ground-state complex formation and, beyond that, the interaction with a quencher (Figure 3). In this case, a short distance between the fluorophore and the quencher is required for the quenching to occur. If the two entities can come in contact, direct electronic interaction can lead to ground state recovery without photon emission [25,26]. When the distance between the fluorophore and the quencher is higher than direct contact, energy transfer such as Förster Resonance Energy Transfer (FRET) can occur [24].

GO can be a super energy acceptor in FRET, due to its broad absorption spectrum (∼300–700 nm) [27]. It has also been demonstrated, both theoretically [28] and experimentally [29], that in contrast to the conventional FRET process with d^−6^ dependency [30], GO can exert an unusual long-range quenching effect, with distances up to 30 nm, proving stronger than a traditional quencher. The surface of GO lends itself to establishing interactions with other molecules, thanks to different linking mechanisms such as hydrogen bonding, electrostatic interactions, or π-stacking. Amongst other things, this latter feature has been exploited to enhance the solubility of poorly water-soluble drugs [31,32,33] that, depending on the molecular structure and geometry, can establish significant π–π interactions. This is also true for aromatic ring-containing fluorophores, such as rhodamines or perylene [34].

When the interacting fluorophore owns a net charge, a stronger quenching effect is observed for cationic dyes than for anionic dyes, due to more intense electrostatic interactions with the GO surface groups. It is easily inferable that pH plays a role, since it influences the protonation/deprotonation degree of hydrogen-containing functional groups on the GO surface and on the fluorescent molecule, as well as their surface charge, thus affecting the extent of attraction or repulsion between these entities. Zeta potential measurements can clearly demonstrate how the negative surface charge of GO particles increases with increasing pH [35]. Temperature is another variable that can affect the quenching process, since it directly influences the Stern–Volmer constant in the case of both static or dynamic collisional quenching [36]. The Stern–Volmer equation (Equation (1)) describes the collisional quenching phenomenon by linking the ratio between the fluorescence intensities in the absence and presence of the quencher (*F*_0_ and *F*, respectively,) to the concentration of the quencher [*Q*].

(1) $$\frac{F_{0}}{F}=1+k_{q}\tau_{0}\left[Q\right]=1+K_{D}[Q]$$

Here, *k_q_* is the bimolecular quenching constant and *τ*_0_ is the lifetime of the fluorophore in the absence of the quencher. The product of these two factors, *k_q_τ*_0_, gives the Stern–Volmer quenching constant *K_D_* in the case of dynamic quenching, or K_SV_ in the case of static quenching [37]. In the case of a dynamic quenching mechanism, the fluorescence decreases proportionally to the collisional frequency between the fluorophore and the quencher, and thus the increasing temperature leads to more efficient quenching because of the higher probability of collisions. When the quenching effect depends on a static quenching, i.e., on the formation of a weakly bound non-fluorescent complex between the fluorophore and the quencher, an increase in temperature can lead to complex dissociation and then to a decrease in the Stern–Volmer constant. In this case, an increased fluorescence intensity is expected at higher temperatures. Stern–Volmer plots obtained from studies on the quenching effect produced by GO on model fluorophores (methylene blue, rhodamine B [17], sulforhodamine B [38], acridine orange, and rhodamine 640 [18]) showed how the main quenching process for these fluorophores is static quenching, as confirmed by lifetime measurements for rhodamine B [10]. Lifetime measurements also showed that, on the contrary, other fluorophores such as carboxytetramethylrhodamine (TAMRA) and tris-(bipyridine)-ruthenium(II) chloride [29], eosin [17] or rhodamine 110 [18] in the presence of GO can undergo a dynamic or mixed quenching effect.

Nonetheless, in some cases GO proved able to exert an enhancing effect on the fluorescence of other molecules. This can be due to peculiar fluorophore characteristics or to experimental variables such as GO concentration or solvent properties. In a work by Yan et al. [39], it was demonstrated that fluorescent systems made of two linked porphyrinic rings can undergo fluorescence enhancement upon coordination with a PEGylated GO (i.e., GO functionalized with polyethylene glycol (PEG) moieties) due to intramolecular charge transfer from one porphyrin ring (connected to the GO surface via π–π interactions) to the other (hanging out far from the GO surface). Qi et al. [40] revealed that GO can enhance rather than quench the fluorescence of fluorophores showing aggregation-induced emission (AIE), such as silole (silacyclopenta-2,4-diene) derivatives. According to the proposed mechanism, the addition of small amounts of GO to 2,5-diethynylsilole-based nanoparticles induces an increase in the particle size, thus promoting aggregation and, consequently, fluorescence. However, after a certain concentration threshold, GO starts to produce the opposite effect, due to the higher probability of one silole nanoparticle being wrapped between two or more GO sheets, thus undergoing significant quenching. These results reveal GO concentration as a crucial factor, as further confirmed by Geng et al. [41]. These authors observed that the fluorescence of water-soluble conjugated polymers with π-conjugated backbones and ionic side chains can be enhanced by the addition of small amounts of GO, thanks to the creation of a hydrophobic environment for the polyelectrolyte backbone. This enhancement effect disappears when the GO concentration increases, because when several GO sheets interact with the polyelectrolyte the quenching effect becomes predominant, overcoming the former enhancement. Additionally, Bapli et al. [42] used several spectroscopic, microscopic and computational techniques to demonstrate that the fluorescence quenching effect produced by GO is strongly solvent-dependent. Peculiar interactions between a fluorophore, GO and their solvent can significantly influence the fluorophore fluorescence intensity, the quantum yield and the lifetime, both in a positive or negative manner, depending on the chosen system. This effect should be carefully taken into account when choosing such a system as a sensing platform.

## 3. Fluorescence-Based GO Sensors for Diagnostics

**Figure 4:**
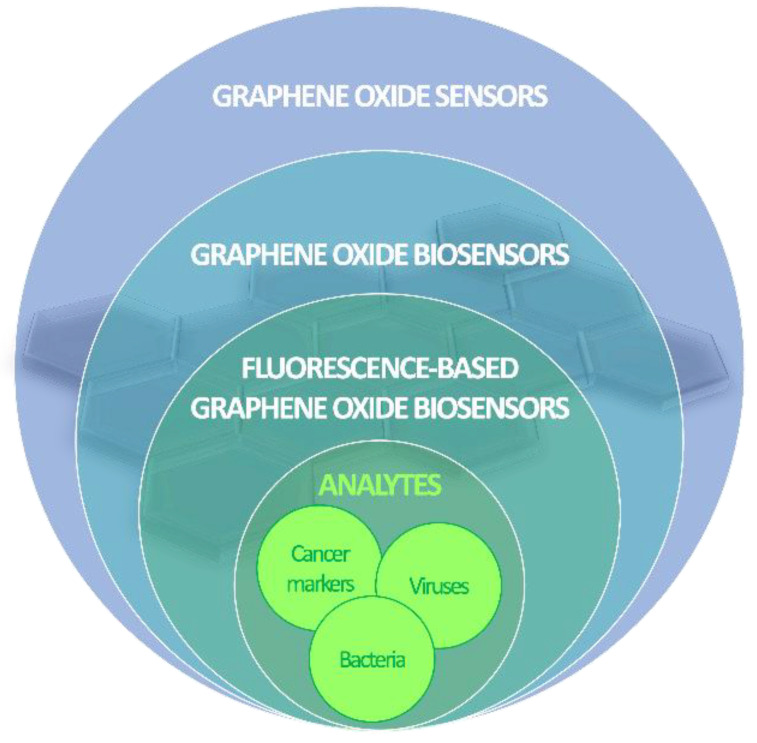
Stacked Venn diagram showing the subset of the sensing applications of GO reviewed in this work.

The peculiar photochemical characteristics of GO make it a suitable sensor component toward a plethora of possible analytes, such as water and VOCs [43], glucose [44], antibiotics [45], and phytohormones [46], to name just a few. Its large surface area, flexibility, thermal stability, optical transparency and ease of production, coupled to nonconductive hydrophilic properties and proneness to functionalization, attracted a wide interest in the biosensing field [47,48]. Several recent advances in the use of GO fluorescence quenching ability for the detection of viruses, bacteria and cancer cells (Figure 4) are reviewed in the following sections.

### 3.1. Diagnosis of Viral Infections

**Figure 5:**
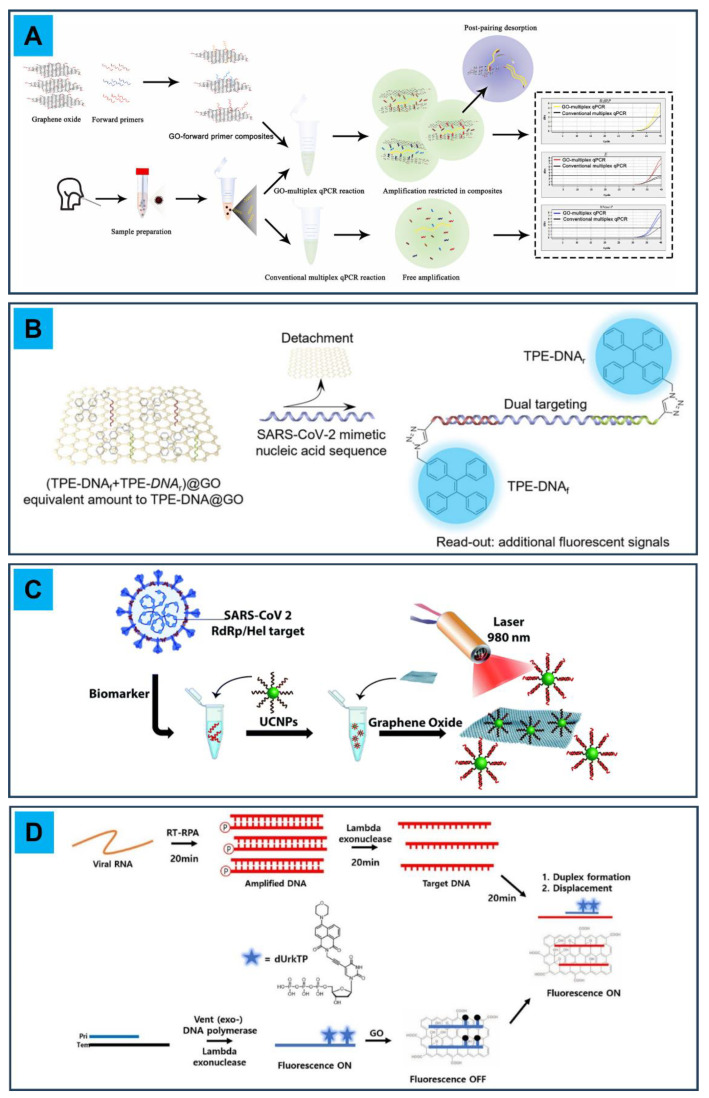
Schematics of GO-based sensors for the detection of SARS-CoV-2. (**A**) Graphical representation of the working principle of GO-forward primer composites in multiplex qPCR. Adapted with permission from Ref. [53]. © 2023 Elsevier B.V. (**B**) (TPE-DNA_f_+TPE-DNA_r_)@GO probing for two binding sites of SARS-CoV-2 mimetic DNA sequence and signal enhancement. Adapted from Ref. [54]. © 2023 by the authors. Published by SCUT, AIEI, and John Wiley & Sons Australia, Ltd. (CC BY 4.0). (**C**) Detection of an oligonucleotide target associated with the RdRp/Hel gene of SARS-CoV-2. Adapted from Ref. [55]. © 2023 by the authors. Published by the Royal Society of Chemistry (CC BY 3.0). (**D**) Combining RPA with an rkDNA–GO system for the detection of COVID-19. Adapted with permission from Ref. [56]. © 2023 Elsevier B.V.

Early detection of viral infections is highly desirable, since it allows for better treatment evaluation and for a prompt, longer therapeutic intervention, thus reducing the likelihood of poor prognoses. This need has been boosted by the recent COVID-19 pandemic event that led to a great demand for the early diagnosis of SARS-CoV-2 infections [49]. Conventional diagnostic systems for viruses rely on cultures, enzyme-linked immunosorbent assay (ELISA) tests on specific immunoglobulins, real-time polymerase chain reaction (PCR), etc. However, improvements in the early diagnosis are still sought in order to offer timely intervention. Virus sensors should comply with selectivity, specificity, sensitivity, stability and cost requirements to be produced on a mass scale and to improve the healthcare chain from disease detection to treatment. Recently, GO has been explored as a potential element in the detection of viral pathogens [50,51]. Its surface can adsorb single-stranded DNA and RNA thanks to π-stacking interactions and hydrogen bonds that can form between the aromatic moieties of the exposed nucleotides and the honeycomb structure of GO. Additionally, GO only rarely adsorbs folded or double-stranded DNA, as showed by ss- and ds-DNA different adsorption kinetics [52]. This ability to adsorb ss-DNA has been exploited to decrease the detection limit of multiplex qPCR analyses for the detection of viral strands such as SARS-CoV-2 [53], according to the principle schematized in Figure 5A. In this context, a highly sequence-specific biosensor was created by Zhang et al. for the point-of-care, one-step detection of SARS-CoV-2-specific nucleic acid sequences (*ORF1ab* or *N* genes) exploiting the combination of aggregation-induced emission luminogen (AIEgen)-labeled oligonucleotide probes with GO nanostructures [54]. Tetraphenylethylene (TPE), a well-known AIEgen, is labeled with DNA and immobilized on the surface of GO nanosheets. Viral nucleic acid detection is performed thanks to a dual “turn-on” mechanism: initially, fluorescence recovery is due to the dissociation of the AIEgen from the GO nanosheet surface in the presence of the target viral nucleic acid; secondly, an enhancement of the AIE-based fluorescent signal is obtained, thanks to the formation of a nucleic acid couple (from single to double strand), able to hinder the intramolecular rotation of the luminogen. This mechanism allows for quick (<1 h) and amplification-free virus detection down to picomolar concentration, thus giving an appreciable preliminary sample screening before PCR confirmation and quantification measurements. Another PCR-free strategy was proposed by Alexaki et al., who developed an oligonucleotide sensor using upconversion nanoparticles (UCNPs) and GO [55]. UCNPs are nanometric particles able to absorb two low-energy photons and to convert them into a high-energy emitted photon, producing an anti-Stokes shift. The proposed UNPCs are composed of a sensitizer ion (Yb^3+^) and an activator ion (Er^3+^) embedded in a lattice (NaYF_4_) whose surface is decorated with synthetic oligonucleotides. In the presence of GO, the oligonucleotide aromatic moieties establish π–π interactions with the GO surface, leading to fluorescence quenching of the particles. When the SARS-CoV-2 oligonucleotide target is present, hybridization of bases takes place, the particles no longer adsorb on the GO surface and the fluorescence signal is recovered. This system proved valid down to femtomolar concentrations of the target. Unlike PCR, which requires time and equipment for gene amplification, isothermal amplification such as recombinase polymerase amplification (RPA) is suitable as a point-of-care detection method, since it requires only about 20 min to sufficiently amplify a target gene for detection. For the detection of the *N* gene of SARS-CoV-2, Choi et al. combined RPA with a turn-on fluorescent rkDNA-GO probe system in order to improve selectivity, thanks to the probe, and sensitivity, thanks to the amplification step, thus overcoming the limitations of the two individual techniques [56]. This method allowed for the quick (about 1 h) and femtomolar-sensitive detection of the target, a limit further improvable with longer (about 1.6 h) amplification times. The cited GO-based sensors for SARS-CoV-2 detection are schematized in Figure 5.

Besides SARS-CoV-2, fluorescence quenching GO-based sensors can be exploited for the rapid detection of other influenza viruses. Moreover, another functional diagnostic tool offered by fluorescence-based GO sensors is multiplexed analysis. This kind of strategy allows for the simultaneous detection of two or more targets in a single measurement. Such a strategy proves valid when applied not only to the detection of multiple analytes but also to the discrimination of different variations of a single analyte, such as viruses subtypes. Orthomyxoviridae is a family of influenza viruses considered a serious threat to public health owing to their wide host breadth, rapid mutation, and aptitude to cause epidemics or even pandemics. They possess a single-stranded negative-sense RNA genome and are classified in genera on the basis of their core structural proteins: genera A, B and C can infect humans. The A type can be further categorized into different subtypes based on the main surface antigens, such as neuraminidase (N) or hemagglutinin (H). Jeong et al. designed a fluorescein amidite FAM-labeled DNA probe, complementary to the hemagglutinin (H) gene sequence of the target influenza virus strain H3N2 [57]. During the RT-PCR for amplification of the H gene, the subtype specific primers are elongated. The 5′ to 3′ exonuclease activity of Taq polymerase hydrolyzes the H gene-bound FAM-DNA probe, so that the FAM fluorophore is released. As soon as the PCR product meets GO, the free FAM produces a detectable fluorescent signal. When the target influenza viral RNA is not present, PCR would produce no product and the FAM-DNA probe would remain intact. Since DNA easily interacts with GO, the FAM fluorescence is quenched and no signal is detected. Picogram sensitivity was reached, and good selectivity towards the target viral strain was achieved. Similarly, Wang et al. demonstrated how GO can be used to produce a rapid, sensitive, and economical double detection system for the two influenza viruses H1N1 and H5N1 in order to systematically screen both pathogenic subtypes [58]. While H1N1 contains genes that facilitate human infection, H5N1 does not transmit efficiently amongst humans. Since the two strains, which produce identical symptoms in humans, have high genetic compatibility and reassortment ability, this kind of double screening could provide an early alert for potential epidemics. In the cited study, a FRET-based aptamer sensor able to detect HA from both H5N1 and H1N1 was proposed. The FAM-labeled aptamer of HA from H5N1 and 6-carboxyl-X-rhodamine (ROX)-labeled aptamer of HA from H1N1 were selected as the energy donors, while GO acted as the energy acceptor. Thanks to the addition of DNase I for amplification, the detection limits of H5N1 HA and H1N1 HA were taken from 20.17 and 8.22 ng/mL (without DNase I) down to 0.733 and 0.427 ng/mL (with DNase I), respectively.

The fluorescence quenching property of GO can be successfully exploited to detect other viruses besides influenza viruses, causing fatal illness in humans. The use of a FAM-labeled detection probe loaded by GO in combination with rolling circle amplification (RCA), which is an isothermal amplification method where a polymerase extends the length of a DNA molecule by several orders of magnitude using a circular primer as the template, proved effective for detecting the Ebola virus (EBOV) [59]. More recently, Fu et al. described a supramolecular sensor array consisting of GO-based fluorogenic peptide probes for the differential sensing of the Ebola virus [60], according to the differential receptor assays concept [61]. Three peptide probes labeled with 5-carboxytetramethylrhodamine (5-TAMRA) were synthesized to self-assemble with GO. The probe was able to unspecifically bind the Ebola as well as the Marburg virus (MARV), which has analogous capsid protein components to EBOV, and the receptor-extensive vesicular stomatitis virus (VSV). However, the fluorescence recovery upon GO binding was different between the different species, thus allowing differential sensing of the viruses using principal components analysis (PCA). PCA is a multivariate technique for the analysis of quantitative data; it exploits multivariate statistical dimensional orthogonal linear transformation for the extraction of features or attributes from a large amount of data signals by reducing the dimensionality of the data from multivariable problems. PCA, coupled to the sensor array concept applied to the GO fluorescence quenching ability, allowed for the recognition of complex viruses expressing different capsid proteins, even with weakly specific peptide probes.

Mosquito-borne viruses such as Zika (ZIKV) and Dengue (DENV) viruses can also be detected, thanks to GO-based sensors. They both belong to the genus Flavivirus and their diffusion is expanding, due to the globalization of transport and global warming, which are widening the spread and habitat of Aedes mosquitoes [62], responsible for the transmission of these viruses. Modern trends aim at the simultaneous diagnosis of the two viruses [63], because a rapid diagnosis of ZIKV and DENV viruses serotype in the acute phase would prevent global transmission of the viruses and allow for efficient treatment of the patients, particularly because of complications due to the possible effects of cross-reactive flavivirus antibodies [64]. In this context, Lee et al. proposed a new molecular diagnostic strategy for multiplexed sensing of flaviviruses using biosensors based on the combination of peptide nucleic acid (PNA), a non-degradable DNA mimic, and GO, possibly combined with loop-mediated isothermal amplification (LAMP) [65]. The aim of their work was the discrimination of the target genome of ZIKV from DENV and the multiplexed detection of DENV serotypes 1 to 4 (DENVs), thanks to GO-based fluorometric biosensors with sequence-specific PNA probes. To validate the sensor, synthetic single-stranded loop DNA sequences for ZIKV and DENVs were used, and the limit of detection (LOD) was found in a range of 2.1 to 5.9 nM, which is suitable for the application of the PNA/GO-based detection system to the virus amplicon. The ability for target differentiation between ZIKV and DENV using the PNA probe mixture was also proved, as well as its multiplexing ability, by using mixtures of targets. Amplification was performed to demonstrate that in, the presence of virus amplicons, the PNA/GO sensor shows notable fluorescence signals compared to the initial low-concentrated virus sample, while in the case of non-target LAMP amplicon controls, no significant fluorescence was detected after the addition of the PNA/GO sensor. Sensitivity of overall detection assays for ZIKV and DENVs was estimated in the range of 2.1 × 10^1^–5.1 × 10^2^ focus forming units per milliliter.

Multiplexed detection based on fluorescence quenching by GO would also be an ideal approach for screening the DNAs of different hepatitis viruses (HV), since viral hepatitis can be caused by five viruses (HV A, B, C, D, and E). Very recently, Guo et al. combined the multi-color fluorescence properties of CdSe/ZnS quantum dots (QDs) with the broad-spectrum fluorescence quenching ability of GO to construct a system based on FRET, wherein the QDs are used as energy donors and GO plays the role of the energy acceptor [66]. Three water-soluble CdSe/ZnS QDs with different maximum emission wavelengths (525 nm, 585 nm and 632 nm) were modified by using complementary ssDNA strands of HV A, B and C DNA, through a cross-linking reaction. The adsorption of QDs on the GO surface produces FRET-induced quenching of their fluorescence. In the presence of DNA belonging to HV A, B and C, the hybridization reaction takes place on the surface of the complementary ssDNA ligand-modified QDs; due to the scarce adsorption of dsDNA on GO, FRET fails and QDs fluorescence is restored, allowing for the multicolor discrimination of the different viruses.

### 3.2. Detection of Bacteria

The increasing global impact of bacterial pathogen resistance is becoming the cause of many life-threatening infections, posing a significant problem of morbidity and mortality around the world. Estimates show that millions of deaths are due to pathogenic bacterial resistance, even more than HIV/AIDS or malaria [67,68,69]. Sometimes, even a single bacterial cell poses a threat to human health [70]. Hence, finding alternatives against pathogenic bacterial resistance is becoming an urgent need. In this context, the detection of various bacterial pathogens with high sensitivity and selectivity is important, and the early detection of these microorganisms, immediately after the onset of the symptoms, can provide a better perspective for human health in terms of therapy success and containment of the infection [71]. Similarly, since many pathogens are responsible for loss of food and agricultural products, exerting possible effects on the economic system of a country, it is also important to develop sensitive sensors to detect harmful pathogens in the environment, in order to prevent diseases through on-time treatment or protection. Existing conventional detection techniques, include the culturing or colony-counting method, polymerase chain reaction (PCR)-based analysis with an additional enrichment step, the combination of colony-counting and PCR methods, ELISA tests, surface plasmon resonance (SPR), piezoelectric quartz crystal techniques, flow cytometry methods, DNA and RNA probes, etc. However, all these advanced techniques have their own drawbacks, such as in the case of the culture method, which is economical but laborious and time-consuming, whereas the ELISA technique is sensitive and rapid but sometimes gives a false output because of cross-reactions, or the PCR technique, which is highly sensitive and specific but not cost-effective [72]. In optical detection systems relying on fluorescent probes or chromogens, when the sample is turbid the detection results are affected by high background signals and poor specificity. Other approaches such as electrochemical sensors can also prove valid; however, they need complicated electrode modifications, and undesired background signals can arise due to various interfering species. Hence, alternative strategies are being explored to address the limitations associated with conventional methods and to develop advanced detection techniques against bacterial pathogens.

Thanks to its large surface area, GO can be exploited to conjugate therapeutic biomolecules via π–π stacking or chemical coupling and to protect them from enzymolysis or biodegradation in the biological systems. Pathogenic bacterial cells can also be adsorbed on the GO surface via protein binding, pili, polysaccharides, and fimbriae, and provide excellent target recognition as well as electrochemical amplification [73]. GO can also exert a bactericidal effect because nanowalls or sharp edges of GO in contact with the bacterial cell membrane can generate a superoxide anion that leads to cell membrane disruption [74]. Furthermore, GO can establish strong interactions with bacterial cells’ lipids and extract a large number of phospholipids, thus causing cell death [75]. The functional groups present in GO can be exploited to impart biorecognition ability to a biosensor. Since GO can be tailored by conjugation of different active biomolecules at the hydroxyl or carboxyl or edges of GO, it can result in a smart material to produce bacteria sensors [76]. In this review, some advanced biosensing applications of GO for the detection of pathogenic bacteria with high sensitivity and selectivity will be discussed.

*Staphylococcus aureus* (*S. aureus*) is a wide-spread pathogen that causes device-related infections, skin, pleuropulmonary, osteoarticular and soft-tissue infections, as well as bacteremia and infective endocarditis, and it has become resistant to β-lactam antibiotics [77]. Although *S. aureus* does not cause serious issues for human health, it can be a problem if not addressed on time. Methicillin-resistant *S. aureus* (MRSA) is a pathogenic bacterium that causes many infectious diseases, both in humans and animals [78]. MRSA is not only resistant to methicillin but also to many other antibiotics, and this resistance led to big clinical challenges in the treatment of hospital and community infections. 

**Figure 6:**
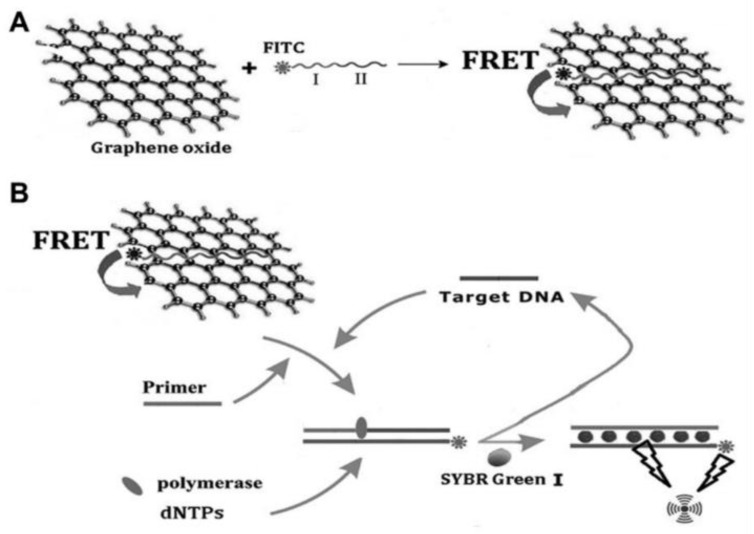
Schematic representation of the GO-based platform for MRSA detection. (**A**) Preparation of GO/probe complexes (I: capture probe, II: signal probe). (**B**) Strand displacement polymerization recycling and synchronous fluorescent signal amplification occurs, leading to an increase in the fluorescence intensity used to quantify the target. Adapted with permission from Ref. [79]. © 2023 Society for Laboratory Automation and Screening (CC BY NC ND 4.0).

Ning et al. designed a novel fluorescent biosensor by combining Klenow fragment (KF)-assisted target recycling amplification with synchronous fluorescence analysis for the detection of MRSA carrying the *mecA* gene [79]. In the absence of the target, the FAM-labeled probe creates π–π interactions with the surface of GO, and its fluorescence is quenched by GO as a result of FRET. As a consequence of the specific recognition between complementary nucleic acid sequences, when the target DNA and primer are present, the probe leaves the GO surface. After the addition of dNTPs and KF, the polymerization reaction that renovates the target sequence for the next round starts, producing a large amount of dsDNA. Afterwards, SYBR Green I is added, and forms a strongly fluorescent DNA-SYBR Green I duplex structure (Figure 6). When target DNA is detected by synchronous fluorescence analysis, since the fluorescence emission of FAM and SYBR Green I completely overlap, fluorescence is significantly enhanced.

This GO-based fluorescence biosensor can detect the *mecA* gene in the range of 1–40 nmol/L, with a lower detection limit of 0.5 nmol/L. Interestingly, when this system is used to analyze pathogenic bacteria, the fluorescence signals are amplified, and a lower detection limit of 3 × 10^2^ CFU/mL is assessed, with a linear range of 10^3^–10^7^ CFU/mL. This biosensor where GO acts as a fluorescence quencher is simple and cost-effective, while providing high sensitivity and selectivity.

In another study by Hunsur Ravikumar et al., a sensitive and selective “ON-OFF-ON” platform based on GO and monoclonal antibody-conjugated quantum dots (mAb-QDs) has been designed to detect micrococcal nuclease (MNase), which is the standard target for *S. aureus* recognition [80]. This probe was designed based on the surface energy transfer mechanism from the weakly coordinated mAb-QDs to GO, wherein mAb-QDs act as donors and GO in proximity serves as the acceptor. In this system, the quenching effect is observed as long as the probe interacts with GO; in the presence of MNase, the stronger affinity between the probe and the analyte hinders the energy transfer to GO, and fluorescence is recovered. The proposed “ON-OFF-ON” detection system was also immobilized on nitrocellulose membranes to produce test strips. The detection limit was found to be 0.3 ng/mL for the fluorescence assay and 0.5 ng/mL for the strips, and it could be used to detect *S. aureus* in real samples.

Another approach consists of sequence-specific recognition of DNA achieved by DNA hybridization of dye-conjugated single-stranded DNA (ssDNA) and the target bacterial DNA, as described above in relation to virus recognition. GO can adsorb dye-conjugated ssDNA probes onto its surface, thus quenching the fluorescence via FRET. In this process, when ssDNA hybridizes with the complementary strand it loses part of its affinity for GO, and the fluorescence of the dye is recovered. The dsDNA shows minor affinity towards GO [52], hence the dsDNA-GO combination exhibits a stronger fluorescence emission than ssDNA-GO. Pang et al. developed a “post-mixing” strategy, where ssDNA labeled with fluorescein isothiocyanate (FITC-DNA) was allowed to hybridize with *S. aureus* DNA before the addition of GO, thus avoiding competition between hybridization and GO absorption [81]. The relative fluorescence intensity resulted to be directly proportional to the *S. aureus* DNA concentration, in the range of 0.0125–3.125 nmol/L^−1^, with a detection limit of 0.00625 nmol/L^−1^ and with excellent sequence selectivity.

Recently, magnetic Fe_3_O_4_ nanoparticle-conjugated GO (MNPs@GO) in combination with aptamer-functionalized lanthanide-doped (NaYF4:Yb/Er) upconversion nanoparticles (UCNPs) were used to develop a biosensor for *S. aureus* detection (MNPs@GO-UCNP). MPNs were modified with a specific aptamer for *S. aureus* and allowed to form π–π bonds with GO. In the absence of *S. aureus*, π–π bonds between aptamer-modified MNPs and GO are intact, and GO cannot be magnetically separated. In the presence of *S. aureus*, MPNs leave the GO surface to bind the bacterial cells, and they can be magnetically separated from free GO. Then, the recovered GO is mixed with UCNPs modified with a non-specific peptide that brings the particles to the GO surface, within FRET distance. The particle fluorescence at 547 nm is then quenched, not only because of FRET but also because the GO absorption band partially overlaps with the UCNPs emission band. This double fluorescence reduction can be exploited to quantify the *S. aureus* concentration. The MNPs@GO-UCNP biosensor is able to detect quantitatively 13 CFU/mL. This biosensor was tested on real chicken meat samples, proving a valid system to monitor bacteria in food samples [82].

*Salmonella* is a Gram-negative facultative intracellular bacterium belonging to the Enterobacteriaceae family. The infections due to this pathogen are mostly related to unhygienic food and wrong food habits including drinking unpasteurized milk and eating raw or undercooked eggs, egg products, meat, poultry, and vegetables. When sufficient *Salmonella* microorganisms enter the stomach, they cause a spectrum of clinical diseases, depending on the infecting bacterial serovar and on the host immune response [83]. Clinically, salmonellosis ranges from the common salmonella gastroenteritis (diarrhea, abdominal cramps, fever, and possible headaches) to enteric fevers (such as typhoid fever) that can be life-threatening when not promptly treated by antibiotic therapy, especially in immunocompromised patients [84]. *Salmonella enterica* serovar *typhimurium* (*S. typhimurium*) is known to be one of the mainly responsible serovars associated with human infections [85], and the early detection of *Salmonella* would prevent several food-borne diseases. In this regard, fluorescence-based, aptamer-modified GO sensors can be also exploited for the detection of this pathogen. This is the case of the paper by Duan et al., where a single-stranded DNA aptamer was chosen from among a set of aptamers with high affinity for *S. typhimurium* individuated by the SELEX procedure from an enriched oligonucleotide pool [86]. Its specificity for *S. typhimurium* was validated using four other food-borne microorganisms as counter-selection targets. The selected FAM-conjugated aptamer was coupled to GO, which induced the fluorescence quenching of FAM. The addition of the target led to the restoration of fluorescence, due to the formation of FAM-aptamer/target complexes, which took the FAM-aptamer away from the GO surface. Under optimal conditions, the assays gave a linear response between 1 × 10^3^ and 1 × 10^8^ CFU/mL, with a LOD of 100 CFU/mL. This sensor was validated using artificially contaminated milk samples.

Another application of a GO-based sensor for the detection of *Salmonella enteritidis* (*S. enteritidis*) in milk samples was developed using the aforementioned properties of GO in combination with hybridization chain reaction (HCR), exploiting the fluorescence quenching effect as well as the discrimination between dsDNA and ssDNA and fluorescence signal amplification effects by HCR [87]. First, ssDNA was prepared as the initiator. For HCR, two metastable DNA hairpins (H1, H2), consisting of a stem, a loop, and a sticky end were selected. The stem forms a stable double helix by base pairing. The sticky single-stranded end is available for hybridization. The H1 sticky end and stem are complementary to part of the target ssDNA. The H2 sticky end is complementary to the loop of H1. The H2 sequence of loop and stem domain that are the same as the target ssDNA sequence are also complementary to the sticky end and stem of H1. In the absence of target bacteria, the sticky ends of the two FAM-labeled hairpins can form π–π interaction with GO, and thus the fluorescence is quenched. In the presence of the target bacteria, the sticky single-stranded part of H1-FAM undergoes hybridization with the bacterial ssDNA and exposes the other section of H1-FAM, which hybridizes with the sticky sequence of H2-FAM. The remaining sequence of H2-FAM opens another H1-FAM, and a long double-stranded DNA is formed, thanks to a repetition of this process. Since in the dsDNA formed by HCR the bases are shielded by the double helix structure, the π–π stacking interaction is prevented and an intense fluorescence signal occurs, revealing the presence of the target bacteria. The LOD achieved by this system was 4.2 × 10^1^ CFU/mL in pure culture, while in an artificially contaminated milk sample *Salmonella* was detected with a LOD of 4.2 × 10^2^ CFU/mL. 

**Figure 7:**
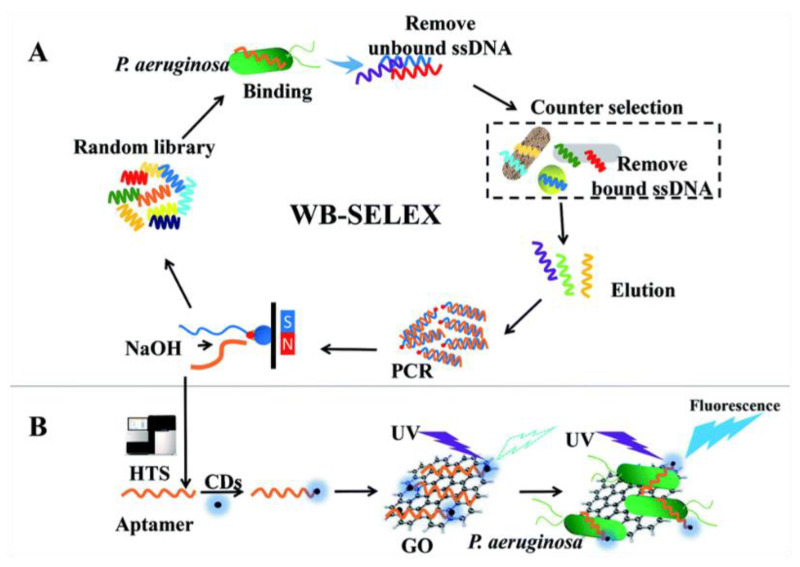
A schematic representation for the development of the fluorescence assay to detect *P. aeruginosa*. (**A**) Whole-bacteria SELEX (WB-SLEX) for selecting aptamer candidates for *P. aeruginosa*. High-throughput sequencing (HTS) was performed for the recognition of aptamer candidates. (**B**) Working principle of aptamer-carbon dots (CDs)/graphene oxide (GO) system for fluorescence detection of *P. aeruginosa*. Adapted with permission from Ref. [89]. © Royal Society of Chemistry 2018 (CC BY NC 3.0).

Other possible bacterial targets include *Pseudomonas* bacteria, which are generally found in soil, water, and vegetation, and can be isolated from the skin, throat, and digestive tract of healthy people. Among the various kinds, *Pseudomonas aeruginosa* (*P. aeruginosa*) is a Gram-negative opportunistic pathogen that often causes various infections in several bodily parts such as blood, lungs, or tissue parts after surgery, constantly finding alternative pathways to elude antibiotic treatments and becoming drug- or multidrug-resistant. It often diffuses in hospital food, sinks, taps, and respiratory equipment. It can spread among patients via contact with infected droplets or by ingestion of contaminated food and water [88]. *P. aeruginosa* is one of the MDR ESKAPE pathogens, which stands for the pathogens *Enterococcus faecium*, *Staphylococcus aureus*, *Klebsiella pneumoniae*, *Acinetobacter baumannii*, *P. aeruginosa*, and *Enterobacter*. Arbapenem-resistant *P. aeruginosa* is considered by WHO as one of the “critical” group of pathogens, which urgently need novel diagnostic and treatment strategies to avoid serious threat to human life. Aptamer-based biosensors can detect pathogenic bacteria with high sensitivity and specificity, and recently aptamers conjugated with carbon dots (CDs) and GO-based biosensors have been developed for culture-independent detection of *P. aeruginosa*. In this system, photoluminescent CDs act as the fluorescent probe, whereas GO anchors aptamers through π–π stacking interaction and acts as a quencher, according to the working principle described above. In the absence of GO, the aptamer-CDs display significant fluorescence. After GO addition, the fluorescence of aptamer-CDs is quenched by GO through FRET. After *P. aeruginosa* is co-incubated with the aptamer-CDs/GO system, the interactions between the bacteria and aptamer-CDs disrupt the π–π stacking interactions, the aptamer-CDs are released from the GO sheet, and fluorescence is significantly recovered (Figure 7). Specificity was tested against six different interfering bacteria. The range for the detection of *P. aeruginosa* was 10^1^–10^7^ CFU/mL with LOD as low as 9 CFU/mL. Hence, this fluorescence biosensor technique can be used to detect *P. aeruginosa* in real water samples to evaluate contamination [89]. The linear detection ranges and limits of detection of the cited sensors are summarized in Table 1.

### 3.3. Detection of Cancer Cells and Biomarkers

Cancer research greatly relies on the development of novel techniques for in vitro imaging of tumor cells, aimed at investigating pathological processes and the efficacy of chemotherapeutic drugs. Fluorescent probes enabling targeted recognition and selective labeling of cancer cells and biomarkers play a key role in addressing research on tumor pathophysiology and therapeutic treatment. In this context, significant progress has been made in the development of novel biosensors exploiting photoluminescence and/or the quenching properties of GO for the detection and imaging of cancer cells and tumor markers. Indeed, more than one substance can be detected on a single biosensor by exploiting the dual role of GO as fluorophore and quencher [90].

One of the first studies exploiting the photoluminescence of GO for cellular imaging was described in 2008 by Sun et al. [91] in a study aimed at the optical identification of cancer cells. They demonstrated that PEGylated GO sheets were photoluminescent and could be covalently conjugated to a specific antibody for B-cell lymphoma cell selective binding. In addition, doxorubicin was adsorbed on GO via π-stacking in order to explore the possibility of selectively transporting an anticancer drug into specific cancer cells by antibody-guided targeting. After the first pioneering studies on this aspect, a rapidly growing number of articles have been dedicated to GO photoluminescence application for cancer cell detection.

In addition, GO has been also combined with metals, such as Au and Ag nanoparticles (NPs), in surface-enhanced Raman scattering (SERS) probes to overcome shortcomings related to photobleaching, autofluorescence, and limited multispectral detection [92]. This approach has been investigated for detecting different kinds of cancer cells and investigating the relevant endocytosis pathway [93,94,95,96]. In addition, different studies demonstrated that this kind of nanoprobe can be functionalized through folic acid covalent conjugation to GO for targeting specific cancer cells with folate receptors (FRs). This approach enables the distinguishing of cells where FRs are overexpressed, such as HeLa cells, and cells where FRs are not over-expressed, such as adenocarcinoma human alveolar basal epithelial cells [97,98].

The high potential of GO fluorescence quenching for cancer diagnostic and therapy has been highlighted over the past few years by an increasing number of articles. In this context, the ability of GO to easily bind single-stranded DNA/RNA fragments (aptamers) through hydrophobic π–π stacking interactions has been often exploited [99]. Indeed, thanks to their small size, high chemical stability, and low immunogenicity, aptamers can bind to their targets with high affinity and specificity. A recent study demonstrated that a biosensor based on fluorescence quenching through FRET can be developed by combining GO with carboxyfluorescein-labeled Sgc8 aptamer (FAM-apt) [100]. This GO-based fluorescent aptasensor was suitable for detecting human acute leukemic lymphoblast cells (CCRF-CEM) in a wide range of concentrations, from 1 × 10^2^ to 1 × 10^7^ cells/mL, with a LOD of 10 cells/mL. CCRF-CEM cells were also detected in the range from 50 to 10^5^ cells by employing a label-free and GO-based aptasensor exploiting cell-triggered cyclic enzymatic signal amplification [101]. Fluorescence analysis demonstrated that this approach enables a detection limit approximately 20 times lower than the LOD of conventional fluorescence aptamer-based sensors without amplification.

**Figure 8:**
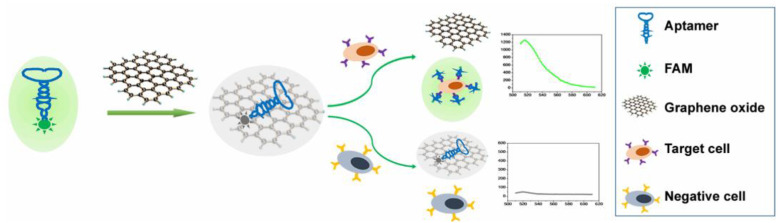
Schematic illustration of the working principle of a GO-based fluorescent aptasensor assay for circulating cancer cell detection. Reproduced from ref. [107]. © 2023 by the authors. Licensee MDPI, Basel, Switzerland (CC BY 4.0).

Other highly sensitive and selective GO-FRET aptasensors based on a fluorophore conjugate to a peptide have been recently developed and employed for detecting breast cancer cells [102], as well as relevant tumor biomarkers [103] such as Mucin 1, which is overexpressed in breast cancer [104]. The huge potential of these biosensors for tumor marker detection has been highlighted also by other articles. As an example, a hybrid ssDNA aptamer was recently demonstrated to be effective in detecting and quantifying the alpha protein biomarker of liver cancer through a concentration-dependent fluorescence recovery mechanism [105]. In addition, this kind of biosensor can be effectively employed for the isolation and detection of different metastatic tumor cells circulating in the bloodstream, including breast, prostate, and colon cancer cells, by covalently attaching specific aptamers to GO platforms [106]. For instance, GO functionalized with 5-carboxyfluorescein (FAM)-labelled W3 aptamer (FAM-W3-GO) was recently applied to detect metastatic colorectal cell lines (LoVo and HCT116) with high sensitivity and a linear increase of fluorescence recovery in a wide range of cell concentrations (0–10^7^ cells/mL) (Figure 8) [107]. This aptasensor was also applied to detect with good reproducibility LoVo cells in human whole blood.

## 4. Conclusions and Perspectives

The peculiar properties of GO such as large surface area, good biocompatibility, water affinity, chemical reactivity, and photochemical behavior, make it a versatile entity in the production of biosensors. Fluorescence-based optical sensors relying on the quenching effect exerted by GO on the probes have the advantage of giving very fast responses and of allowing multiplex detection of different analytes at the same time. In this review, we showed that GO-based sensors can reach low LODs in wide linear detection ranges, along with high specificity and selectivity towards several analytes, and particularly towards viruses, bacteria and cancer markers. 

In the detection of viruses, standard tests are currently based on cell cultures, thus relying upon the evaluation of the cytopathic effect and hemadsorption caused by the pathogen, or on molecular assays based on nucleic acid and PCR systems [108]. Even real-time PCR, which lowers the analysis duration to a few hours, still requires specialist equipment and reagents. GO sensors can fulfill the need for a highly accurate diagnosis, associated with fast analysis, low cost, ease of use and portability.

Bacteria are conventionally detected by laboratory methods (e.g., microscopy and cell culture, immunological methods, biochemical tests, genetic analysis) that often show long processing times, inadequate sensitivity and specificity, and high costs, and that require considerable specialization in terms of equipment and users [109]. Sensors based on the reviewed GO technology can be designed to provide quick, cheap, and reliable tests achieving low LODs and which are able to identify bacteria during an in-field analysis or at the point-of-care, thus avoiding multistep procedures for processing and purification.

Concerning cancer detection, it is evident that an early diagnosis is essential for timely treatment. Unfortunately, some of the conventional diagnostic strategies such as centrifugation, chromatography, and magnetic-activated cell sorting are highly dependent on the operator’s skills, while magnetic resonance imaging, computed tomography, and X-ray radiography are expensive, and the waiting time can be long. Conversely, GO can be used to produce conjugate materials providing efficient reagentless biosensing with high performance in terms of response quickness (seconds to minutes), sensitivity (detection at sub-picomolar-to-micromolar concentrations) and selectivity.

Shortcomings relevant to the preparation and reproducibility of the end product are still being tackled; this material is under continuous investigation to make it more efficient, cheaper, and more reliable in terms of final performances. Moreover, GO is currently being investigated in its doped forms (e.g., with nitrogen, sulfur or chlorine), because the doping by different chemical elements can alter the optical and electronic features of GO, including its own fluorescence and its quenching ability [110]. This means that a plethora of novel GO versions still need to be put to the test for possible sensing applications.

There is no doubt that the use of GO has been constantly widening to reach an ever-broader spectrum of applications, and results obtained so far have made it a main player in the biosensing field. Given the huge demand for point-of-care and laboratory high-performance sensors, it is easy to foresee that many of the current proof-of-concept devices will gain commercial interest on the market in the near future. Forthcoming advances in the chemistry of GO and its derivatives will hopefully aim towards the ultimate theranostic approach, combining in situ biosensing with real-time monitoring and the associated photosensitizing effect for photothermal therapy or drug delivery for disease treatment.

## Figures and Tables

**Table 1:** Linear detection range (LDR) and LOD of GO-based system for bacteria detection.

Target	LDR	LOD	Reference
*S. aureus mecA* gene	1–40 nM	0.5 nM	[79]
*S. aureus*	10^3^–10^7^ CFU/mL	3 × 10^2^ CFU/mL	[79]
*S. aureus* MNase	0.1–10 ng/mL	0.3 ng/mL	[80]
*S. aureus* DNA	0.0125–3.125 nM	0.00625 nM	[81]
*S. aureus*	86–8.6 × 10^7^ CFU/mL	13 CFU/mL	[82]
*S. typhimurium*	1 × 10^3^–1 × 10^8^ CFU/mL	10^2^ CFU/mL	[86]
*S. enteritidis*	4.2 × 10^1^–4.2 × 10^8^ CFU/mL	4.2 × 10^2^ CFU/mL	[87]
*P. aeruginosa*	10^1^–10^7^ CFU/mL	9 CFU/mL	[89]

## Data Availability

This is a review article. Data sharing is not applicable to this article.

## References

[1] Speranza G. (2021). Carbon Nanomaterials: Synthesis, Functionalization and Sensing Applications. Nanomaterials.

[2] Hummers W.S., Offeman R.E. (1958). Preparation of Graphitic Oxide. J. Am. Chem. Soc..

[3] Feng L., Wu L., Qu X., Feng L., Wu L., Qu X. (2013). New Horizons for Diagnostics and Therapeutic Applications of Graphene and Graphene Oxide. Adv. Mater..

[4] Chung C., Kim Y.K., Shin D., Ryoo S.R., Hong B.H., Min D.H. (2013). Biomedical Applications of Graphene and Graphene Oxide. Acc. Chem. Res..

[5] Priyadarsini S., Mohanty S., Mukherjee S., Basu S., Mishra M. (2018). Graphene and Graphene Oxide as Nanomaterials for Medicine and Biology Application. J. Nanostruct. Chem..

[6] Dreyer D.R., Park S., Bielawski C.W., Ruoff R.S. (2009). The Chemistry of Graphene Oxide. Chem. Soc. Rev..

[7] Dideikin A.T., Vul’ A.Y. (2019). Graphene Oxide and Derivatives: The Place in Graphene Family. Front. Phys..

[8] Aliyev E., Filiz V., Khan M.M., Lee Y.J., Abetz C., Abetz V. (2019). Structural Characterization of Graphene Oxide: Surface Functional Groups and Fractionated Oxidative Debris. Nanomaterials.

[9] Choi Y.R., Yoon Y.G., Choi K.S., Kang J.H., Shim Y.S., Kim Y.H., Chang H.J., Lee J.H., Park C.R., Kim S.Y. (2015). Role of Oxygen Functional Groups in Graphene Oxide for Reversible Room-Temperature NO_2_ Sensing. Carbon.

[10] Sajjad S., Khan Leghari S.A., Iqbal A. (2017). Study of Graphene Oxide Structural Features for Catalytic, Antibacterial, Gas Sensing, and Metals Decontamination Environmental Applications. ACS Appl. Mater. Interfaces.

[11] Lee J., Kim J., Kim S., Min D.H. (2016). Biosensors Based on Graphene Oxide and Its Biomedical Application. Adv. Drug Deliv. Rev..

[12] Samota S., Rani R., Chakraverty S., Kaushik A. (2022). Biosensors for Simplistic Detection of Pathogenic Bacteria: A Review with Special Focus on Field-Effect Transistors. Mater. Sci. Semicond. Process.

[13] Ivnitski D., Abdel-Hamid I., Atanasov P., Wilkins E. (1999). Biosensors for Detection of Pathogenic Bacteria. Biosens. Bioelectron..

[14] Kim J., Park S.J., Min D.H. (2017). Emerging Approaches for Graphene Oxide Biosensor. Anal. Chem..

[15] Nurrohman D.T., Chiu N.-F. (2021). A Review of Graphene-Based Surface Plasmon Resonance and Surface-Enhanced Raman Scattering Biosensors: Current Status and Future Prospects. Nanomaterials.

[16] Roy S., Soin N., Bajpai R., Misra D.S., McLaughlin J.A., Roy S.S. (2011). Graphene Oxide for Electrochemical Sensing Applications. J. Mater. Chem..

[17] Liu Y.Y., Liu C.Y., Liu Y.Y. (2011). Investigation on Fluorescence Quenching of Dyes by Graphite Oxide and Graphene. Appl. Surf. Sci..

[18] Povedailo V.A., Ronishenko B.V., Stepuro V.I., Tsybulsky D.A., Shmanai V.V., Yakovlev D.L. (2018). Fluorescence Quenching of Dyes by Graphene Oxide. J. Appl. Spectrosc..

[19] Huang S.T., Shi Y., Li N.B., Luo H.Q. (2012). Fast and Sensitive Dye-Sensor Based on Fluorescein/Reduced Graphene Oxide Complex. Analyst.

[20] Liu D., Wang Q., Chen A., Li Q., Sui L., Jin M. (2021). Ultrafast Dynamics on Fluorescence Quenching of Rhodamine 6G by Graphene Oxide. Luminescence.

[21] Tran L.H., Lee C.W., Kang T.J., Jang S.H. (2016). Graphene Oxide-Mediated Fluorescence Quenching of Green Fluorescent Protein for Biomedical Applications. Bull. Korean Chem. Soc..

[22] Jang H., Lee J., Min D.H. (2014). Graphene Oxide for Fluorescence-Mediated Enzymatic Activity Assays. J. Mater. Chem. B.

[23] Li S., Aphale A.N., Macwan I.G., Patra P.K., Gonzalez W.G., Miksovska J., Leblanc R.M. (2012). Graphene Oxide as a Quencher for Fluorescent Assay of Amino Acids, Peptides, and Proteins. ACS Appl. Mater. Interfaces.

[24] Zheng P., Wu N. (2017). Fluorescence and Sensing Applications of Graphene Oxide and Graphene Quantum Dots: A Review. Chem. Asian J..

[25] Asha Jhonsi M., Nithya C., Kathiravan A. (2014). Probing Electron Transfer Dynamics of Pyranine with Reduced Graphene Oxide. Phys. Chem. Chem. Phys..

[26] Zhao Y., Li K., He Z., Zhang Y., Zhao Y., Zhang H., Miao Z. (2016). Investigation on Fluorescence Quenching Mechanism of Perylene Diimide Dyes by Graphene Oxide. Molecules.

[27] Zhu Y., Cai Y., Xu L., Zheng L., Wang L., Qi B., Xu C. (2015). Building An Aptamer/Graphene Oxide FRET Biosensor for One-Step Detection of Bisphenol A. ACS Appl. Mater. Interfaces.

[28] Swathi R.S., Sebastian K.L. (2009). Long Range Resonance Energy Transfer from a Dye Molecule to Graphene Has (Distance)-4 Dependence. J. Chem. Phys..

[29] Wu X., Xing Y., Zeng K., Huber K., Zhao J.X. (2018). Study of Fluorescence Quenching Ability of Graphene Oxide with a Layer of Rigid and Tunable Silica Spacer. Langmuir.

[30] Schaufele F., Demarco I., Day R.N. (2005). FRET Imaging in the Wide-Field Microscope. Molecular Imaging: FRET Microscopy and Spectroscopy.

[31] Islam M.S., Renner F., Azizighannad S., Mitra S. (2020). Direct Incorporation of Nano Graphene Oxide (NGO) into Hydrophobic Drug Crystals for Enhanced Aqueous Dissolution. Colloids Surf. B Biointerfaces.

[32] Pereira De Sousa I., Buttenhauser K., Suchaoin W., Partenhauser A., Perrone M., Matuszczak B., Bernkop-Schnürch A. (2016). Thiolated Graphene Oxide as Promising Mucoadhesive Carrier for Hydrophobic Drugs. Int. J. Pharm..

[33] Wei L., Lu Z., Ji X., Jiang Y., Ma L. (2021). Self-Assembly of Hollow Graphene Oxide Microcapsules Directed by Cavitation for Loading Hydrophobic Drugs. ACS Appl. Mater. Interfaces.

[34] Zhang H.-S., Dong X.-M., Zhang Z.-C., Zhang Z.-P., Ban C.-Y., Zhou Z., Song C., Yan S.-Q., Xin Q., Liu J.-Q. (2022). Co-Assembled Perylene/Graphene Oxide Photosensitive Heterobilayer for Efficient Neuromorphics. Nat. Commun..

[35] Hu X., Yu Y., Hou W., Zhou J., Song L. (2013). Effects of Particle Size and pH Value on the Hydrophilicity of Graphene Oxide. Appl. Surf. Sci..

[36] Srisantitham S., Sukwattanasinitt M., Unarunotai S. (2018). Effect of pH on Fluorescence Quenching of Organic Dyes by Graphene Oxide. Colloids Surf. A Physicochem. Eng. Asp..

[37] Lakowicz J.R. (2006). Principles of Fluorescence Spectroscopy.

[38] Ray K., Nakahara H. (2002). Adsorption of Sulforhodamine Dyes in Cationic Langmuir-Blodgett Films: Spectroscopic and Structural Studies. J. Phys. Chem. B.

[39] Yan X., Niu G., Lin J., Jin A.J., Hu H., Tang Y., Zhang Y., Wu A., Lu J., Zhang S. (2015). Enhanced Fluorescence Imaging Guided Photodynamic Therapy of Sinoporphyrin Sodium Loaded Graphene Oxide. Biomaterials.

[40] Qi C.X., Li H., Lam J.W.Y., Yuan X., Wei J., Tang B.Z., Zhang H., Qi X.Y., Li H., Yuan X.T. (2012). Graphene Oxide as a Novel Nanoplatform for Enhancement of Aggregation-Induced Emission of Silole Fluorophores. Adv. Mater..

[41] Geng J., Zhou L., Liu B. (2013). Graphene Oxide Enhanced Fluorescence of Conjugated Polyelectrolytes with Intramolecular Charge Transfer Characteristics. Chem. Commun..

[42] Bapli A., Kumar Gautam R., Seth S., Jana R., Pandit S., Seth D., Bapli A., Gautam R.K., Jana R., Pandit S. (2020). Graphene Oxide as an Enhancer of Fluorescence. Chem. Asian J..

[43] Toda K., Furue R., Hayami S. (2015). Recent Progress in Applications of Graphene Oxide for Gas Sensing: A Review. Anal. Chim. Acta.

[44] Thakur S., Verma A., Alsanie W.F., Christie G., Thakur V.K. (2022). On the Graphene and Its Derivative Based Polymer Nanocomposites for Glucose Sensing. Mater. Lett..

[45] Fu L., Mao S., Chen F., Zhao S., Su W., Lai G., Yu A., Lin C. (2022). Te Graphene-Based Electrochemical Sensors for Antibiotic Detection in Water, Food and Soil: A Scientometric Analysis in CiteSpace (2011–2021). Chemosphere.

[46] Yang M., Wang L., Lu H., Dong Q., Li H., Liu S. (2022). Graphene and Graphene-like Carbon Nanomaterials-Based Electrochemical Biosensors for Phytohormone Detection. Carbon Lett..

[47] Zeng L., Cao S., Yin H., Xiong J., Lin D., Zeng L., Cao S., Yin H., Xiong J., Lin D. (2018). Graphene Oxide-Based Biosensors.

[48] Peña-Bahamonde J., Nguyen H.N., Fanourakis S.K., Rodrigues D.F. (2018). Recent Advances in Graphene-Based Biosensor Technology with Applications in Life Sciences. J. Nanobiotechnol..

[49] Luo Z., Ang M.J.Y., Chan S.Y., Yi Z., Goh Y.Y., Yan S., Tao J., Liu K., Li X., Zhang H. (2020). Combating the Coronavirus Pandemic: Early Detection, Medical Treatment, and a Concerted Effort by the Global Community. Research.

[50] Vermisoglou E., Panáček D., Jayaramulu K., Pykal M., Frébort I., Kolář M., Hajdúch M., Zbořil R., Otyepka M. (2020). Human Virus Detection with Graphene-Based Materials. Biosens. Bioelectron..

[51] Salama A.M., Yasin G., Zourob M., Lu J. (2022). Fluorescent Biosensors for the Detection of Viruses Using Graphene and Two-Dimensional Carbon Nanomaterials. Biosensors.

[52] Huang P.J.J., Liu J. (2013). Separation of Short Single- and Double-Stranded DNA Based on Their Adsorption Kinetics Difference on Graphene Oxide. Nanomaterials.

[53] Zeng Y., Zhou L., Yang Z., Yu X., Song Z., He Y. (2022). High Sensitivity SARS-CoV-2 Detection Using Graphene Oxide-Multiplex QPCR. Anal. Chim. Acta.

[54] Zhang Q., Yin B., Hao J., Ma L., Huang Y., Shao X., Li C., Chu Z., Yi C., Hong S. (2022). An AIEgen/Graphene Oxide Nanocomposite (AIEgen@GO)-Based Two-Stage “Turn-on” Nucleic Acid Biosensor for Rapid Detection of SARS-CoV-2 Viral Sequence. Aggregate.

[55] Alexaki K., Kyriazi M.E., Greening J., Taemaitree L., El-Sagheer A.H., Brown T., Zhang X., Muskens O.L., Kanaras A.G. (2022). A SARS-Cov-2 Sensor Based on Upconversion Nanoparticles and Graphene Oxide. RSC Adv..

[56] Choi M.H., Lee J., Seo Y.J. (2021). Combined Recombinase Polymerase Amplification/RkDNA–Graphene Oxide Probing System for Detection of SARS-CoV-2. Anal. Chim. Acta.

[57] Jeong S., Kim D.M., An S.Y., Kim D.H., Kim D.E. (2018). Fluorometric Detection of Influenza Viral RNA Using Graphene Oxide. Anal. Biochem..

[58] Wang Z., Zhao Q., Huang M., Duan Y., Li F., Wang T. (2022). Dual Detection of Hemagglutinin Proteins of H5N1 and H1N1 Influenza Viruses Based on FRET Combined With DNase I. Front. Microbiol..

[59] Wen J., Li W., Li J., Tao B., Xu Y., Li H., Lu A., Sun S. (2016). Study on Rolling Circle Amplification of Ebola Virus and Fluorescence Detection Based on Graphene Oxide. Sens. Actuators B Chem..

[60] Fu M.Q., Wang X.C., Dou W.T., Chen G.R., James T.D., Zhou D.M., He X.P. (2020). Supramolecular Fluorogenic Peptide Sensor Array Based on Graphene Oxide for the Differential Sensing of Ebola Virus. Chem. Commun..

[61] Wright A.T., Anslyn E.V. (2006). Differential Receptor Arrays and Assays for Solution-Based Molecular Recognition. Chem. Soc. Rev..

[62] Yeom J. (2017). Current Status and Outlook of Mosquito-Borne Diseases in Korea. J. Korean Med. Assoc..

[63] Park G., Park H., Park S.-C., Jang M., Yoon J., Ahn J.-H., Lee T. (2023). Recent Developments in DNA-Nanotechnology-Powered Biosensors for Zika/Dengue Virus Molecular Diagnostics. Nanomaterials.

[64] Langerak T., Mumtaz N., Tolk V.I., Van Gorp E.C.M., Martina B.E., Rockx B., Koopmans M.P.G. (2019). The Possible Role of Cross-Reactive Dengue Virus Antibodies in Zika Virus Pathogenesis. PLoS Pathog..

[65] Lee J.S., Kim J., Shin H., Min D.H. (2020). Graphene Oxide-Based Molecular Diagnostic Biosensor for Simultaneous Detection of Zika and Dengue Viruses. 2D Mater..

[66] Guo J., Zhang H., Yang J., Zhang Y., Wang J., Yan G. (2023). SsDNA-QDs/GO Multicolor Fluorescence System for Synchronous Screening of Hepatitis Virus DNA. Arab. J. Chem..

[67] Murray C.J., Ikuta K.S., Sharara F., Swetschinski L., Robles Aguilar G., Gray A., Han C., Bisignano C., Rao P., Wool E. (2022). Global Burden of Bacterial Antimicrobial Resistance in 2019: A Systematic Analysis. Lancet.

[68] Limmathurotsakul D., Dunachie S., Fukuda K., Feasey N.A., Okeke I.N., Holmes A.H., Moore C.E., Dolecek C., van Doorn H.R., Shetty N. (2019). Improving the Estimation of the Global Burden of Antimicrobial Resistant Infections. Lancet Infect. Dis..

[69] Davies J., Davies D. (2010). Origins and Evolution of Antibiotic Resistance. Microbiol. Mol. Biol. Rev..

[70] Kaper J.B., Nataro J.P., Mobley H.L.T. (2004). Pathogenic Escherichia Coli. Nat. Rev. Microbiol..

[71] Pang Q., Lou D., Li S., Wang G., Qiao B., Dong S., Ma L., Gao C., Wu Z. (2020). Smart Flexible Electronics-Integrated Wound Dressing for Real-Time Monitoring and On-Demand Treatment of Infected Wounds. Adv. Sci..

[72] Anand T., Narasa Raju T.A., Vishnu C., Venkateswar Rao L., Sharma G. (2001). Development of Dot-ELISA for the Detection of Human Rotavirus Antigen and Comparison with RNA-PAGE. Lett. Appl. Microbiol..

[73] Nair S., Kumar V., Kumar R., Jain V.K., Nagpal S. (2022). Electrostatic Graphene Oxide-Based Biosensor for Rapid Direct Detection of *E. coli*. Int. J. Mater. Res..

[74] Linklater D.P., Baulin V.A., Juodkazis S., Ivanova E.P. (2018). Mechano-Bactericidal Mechanism of Graphene Nanomaterials. Interface Focus..

[75] Tu Y., Lv M., Xiu P., Huynh T., Zhang M., Castelli M., Liu Z., Huang Q., Fan C., Fang H. (2013). Destructive Extraction of Phospholipids from Escherichia Coli Membranes by Graphene Nanosheets. Nat. Nanotechnol..

[76] Chang J., Mao S., Zhang Y., Cui S., Zhou G., Wu X., Yang C.H., Chen J. (2013). Ultrasonic-Assisted Self-Assembly of Monolayer Graphene Oxide for Rapid Detection of Escherichia Coli Bacteria. Nanoscale.

[77] Tong S.Y.C., Davis J.S., Eichenberger E., Holland T.L., Fowler V.G. (2015). *Staphylococcus aureus* Infections: Epidemiology, Pathophysiology, Clinical Manifestations, and Management. Clin. Microbiol. Rev..

[78] Lee A.S., De Lencastre H., Garau J., Kluytmans J., Malhotra-Kumar S., Peschel A., Harbarth S. (2018). Methicillin-Resistant *Staphylococcus aureus*. Nat. Rev. Dis. Primers.

[79] Ning Y., Gao Q., Zhang X., Wei K., Chen L. (2016). A Graphene Oxide-Based Sensing Platform for the Determination of Methicillin-Resistant *Staphylococcus aureus* Based on Strand-Displacement Polymerization Recycling and Synchronous Fluorescent Signal Amplification. J. Biomol. Screen..

[80] Hunsur Ravikumar C., Ira Gowda M., Balakrishna R.G. (2019). An “OFF–ON” Quantum Dot–Graphene Oxide Bioprobe for Sensitive Detection of Micrococcal Nuclease of *Staphylococcus aureus*. Analyst.

[81] Pang S., Gao Y., Li Y., Liu S., Su X. (2013). A Novel Sensing Strategy for the Detection of *Staphylococcus aureus* DNA by Using a Graphene Oxide-Based Fluorescent Probe. Analyst.

[82] Chen M., Song Y., Han L., Zhou D., Wang Y., Pan L., Tu K. (2022). An Ultrasensitive Upconversion Fluorescence Aptasensor Based on Graphene Oxide Release and Magnetic Separation for *Staphylococcus aureus* Detection. Food Anal. Methods.

[83] Griffin A.J., McSorley S.J. (2011). Development of Protective Immunity to Salmonella, a Mucosal Pathogen with a Systemic Agenda. Mucosal Immunol..

[84] Giannella R.A. (1996). Salmonella. Medical Microbiology.

[85] Cody S.H., Abbott S.L., Marfin A.A., Schulz B., Wagner P., Robbins K., Mohle-Boetani J.C., Vugia D.J. (1999). Two Outbreaks of Multidrug-Resistant Salmonella Serotype Typhimurium DT104 Infections Linked to Raw-Milk Cheese in Northern California. JAMA.

[86] Duan Y.F., Ning Y., Song Y., Deng L. (2014). Fluorescent Aptasensor for the Determination of Salmonella Typhimurium Based on a Graphene Oxide Platform. Microchim. Acta.

[87] Yu S., Xu Q., Huang J., Yi B., Aguilar Z.P., Xu H. (2021). Rapid and Sensitive Detection of Salmonella in Milk Based on Hybridization Chain Reaction and Graphene Oxide Fluorescence Platform. J. Dairy Sci..

[88] Das K., Abrol S., Verma R., Annapragada H., Katiyar N., Senthilkumar M. (1996). Pseudomonas. Beneficial Microbes in Agro-Ecology: Bacteria and Fungi.

[89] Wang H., Chi Z., Cong Y., Wang Z., Jiang F., Geng J., Zhang P., Ju P., Dong Q., Liu C. (2018). Development of a Fluorescence Assay for Highly Sensitive Detection of Pseudomonas Aeruginosa Based on an Aptamer-Carbon Dots/Graphene Oxide System. RSC Adv..

[90] Parvin N., Jin Q., Wei Y., Yu R., Zheng B., Huang L., Zhang Y., Wang L., Zhang H., Gao M. (2017). Few-Layer Graphdiyne Nanosheets Applied for Multiplexed Real-Time DNA Detection. Adv. Mater..

[91] Sun X., Liu Z., Welsher K., Robinson J.T., Goodwin A., Zaric S., Dai H. (2008). Nano-Graphene Oxide for Cellular Imaging and Drug Delivery. Nano Res..

[92] Ock K., Jeon W.I., Ganbold E.O., Kim M., Park J., Seo J.H., Cho K., Joo S.-W., Lee S.Y. (2012). Real-Time Monitoring of Glutathione-Triggered Thiopurine Anticancer Drug Release in Live Cells Investigated by Surface-Enhanced Raman Scattering. Anal. Chem..

[93] Liu Q., Wei L., Wang J., Peng F., Luo D., Cui R., Niu Y., Qin X., Liu Y., Sun H. (2012). Cell Imaging by Graphene Oxide Based on Surface Enhanced Raman Scattering. Nanoscale.

[94] Huang J., Zong C., Shen H., Liu M., Chen B., Ren B., Zhang Z. (2012). Mechanism of Cellular Uptake of Graphene Oxide Studied by Surface-Enhanced Raman Spectroscopy. Small.

[95] Manikandan M., Nasser Abdelhamid H., Talib A., Wu H.F. (2014). Facile Synthesis of Gold Nanohexagons on Graphene Templates in Raman Spectroscopy for Biosensing Cancer and Cancer Stem Cells. Biosens. Bioelectron..

[96] Yim D.B., Kang H., Jeon S.J., Kim H.I., Yang J.K., Kang T.W., Lee S., Choo J., Lee Y.S., Kim J.W. (2015). Graphene Oxide-Encoded Ag Nanoshells with Single-Particle Detection Sensitivity towards Cancer Cell Imaging Based on SERRS. Analyst.

[97] Hu C., Liu Y., Qin J., Nie G., Lei B., Xiao Y., Zheng M., Rong J. (2013). Fabrication of Reduced Graphene Oxide and Sliver Nanoparticle Hybrids for Raman Detection of Absorbed Folic Acid: A Potential Cancer Diagnostic Probe. ACS Appl. Mater. Interfaces.

[98] Liu Z., Guo Z., Zhong H., Qin X., Wan M., Yang B. (2013). Graphene Oxide Based Surface-Enhanced Raman Scattering Probes for Cancer Cell Imaging. Phys. Chem. Chem. Phys..

[99] Sekhon S.S., Kaur P., Kim Y.H., Sekhon S.S. (2021). 2D Graphene Oxide–Aptamer Conjugate Materials for Cancer Diagnosis. 2D Mater. Appl..

[100] Tan J., Lai Z., Zhong L., Zhang Z., Zheng R., Su J., Huang Y., Huang P., Song H., Yang N. (2018). A Graphene Oxide-Based Fluorescent Aptasensor for the Turn-on Detection of CCRF-CEM. Nanoscale Res. Lett..

[101] Xiao K., Liu J., Chen H., Zhang S., Kong J. (2017). A Label-Free and High-Efficient GO-Based Aptasensor for Cancer Cells Based on Cyclic Enzymatic Signal Amplification. Biosens. Bioelectron..

[102] Wang X., Han Q., Yu N., Li J., Yang L., Yang R., Wang C. (2015). Aptamer–Conjugated Graphene Oxide–Gold Nanocomposites for Targeted Chemo-Photothermal Therapy of Cancer Cells. J. Mater. Chem. B.

[103] Shi H., Zhang B., Liu S., Tan C., Tan Y., Jiang Y. (2018). A New Strategy Involving the Use of Peptides and Graphene Oxide for Fluorescence Turn-on Detection of Proteins. Sensors.

[104] Ding Y., Ling J., Wang H., Zou J., Wang K., Xiao X., Yang M. (2015). Fluorescent Detection of Mucin 1 Protein Based on Aptamer Functionalized Biocompatible Carbon Dots and Graphene Oxide. Anal. Methods.

[105] Wang C.F., Wang Z.G., Sun X.Y., Chen M.J., Lv Y.K. (2018). An Ultrasensitive Fluorescent Aptasensor for Detection of Cancer Marker Proteins Based on Graphene Oxide–SsDNA. RSC Adv..

[106] Viraka Nellore B.P., Kanchanapally R., Pramanik A., Sinha S.S., Chavva S.R., Hamme A., Ray P.C. (2015). Aptamer-Conjugated Graphene Oxide Membranes for Highly Efficient Capture and Accurate Identification of Multiple Types of Circulating Tumor Cells. Bioconjug. Chem..

[107] Chen H., Zhang S., Hsiao Y.C., Wang Q., Yu J.S., Li W. (2022). Graphene Oxide and Fluorescent-Aptamer-Based Novel Aptasensors for Detection of Metastatic Colorectal Cancer Cells. Polymers.

[108] Ribeiro B.V., Cordeiro T.A.R., Oliveira e Freitas G.R., Ferreira L.F., Franco D.L. (2020). Biosensors for the Detection of Respiratory Viruses: A Review. Talanta Open.

[109] Ahmed A., Rushworth J.V., Hirst N.A., Millner P.A. (2014). Biosensors for Whole-Cell Bacterial Detection. Clin. Microbiol. Rev..

[110] Xiao X., Zhang Y., Zhou L., Li B., Gu L. (2022). Photoluminescence and Fluorescence Quenching of Graphene Oxide: A Review. Nanomaterials.

